# Seaweed Foliar Biostimulants Improve Growth and Phytochemicals of Thai Basil (*Ocimum basilicum* L.) in a Plant Factory

**DOI:** 10.3390/plants14213271

**Published:** 2025-10-26

**Authors:** Vu Phong Lam, Gwonjeong Bok, Dao Nhan Loi, Manh Cuong Do, Jongseok Park

**Affiliations:** 1 Department of Horticultural Science, Chungnam National University, Daejeon 34134, Republic of Korea; phonglamdhtaybac@gmail.com; 2 Faculty of Agriculture and Forestry, Tay Bac University, Son La 360000, Vietnam; daonhanloi@gmail.com; 3 Glocal University Project Team, Sunchon National University, Suncheon 57922, Republic of Korea; bokc4578@scnu.ac.kr; 4 Department of Bio-AI Convergence, Chungnam National University, Daejeon 34134, Republic of Korea; 5 Institute of Theoretical and Applied Research, Duy Tan University, Hanoi 100000, Vietnam; 6 College of Medicine and Pharmacy, Duy Tan University, Da Nang 550000, Vietnam

**Keywords:** antioxidant activity, chlorophyll, concentration, flavonoid, photosynthetic parameters

## Abstract

This study aimed to identify the optimal concentration of seaweed extract (SE) for enhancing growth, photosynthetic traits, antioxidant activity, and bioactive compound accumulation in Thai basil (*Ocimum basilicum* L.) plants cultivated in a fully controlled plant factory. Basil plants were foliar-sprayed twice weekly with five SE concentrations (0.5, 1.0, 1.5, 2.0, and 2.5 mL·L^−1^), while untreated plants served as controls. After 28 days of transplanting, plant growth parameters, photosynthetic parameters, chlorophyll pigments, antioxidant activity, and the concentrations of phenolic acids and rosmarinic acid (RA) were analyzed. Moderate SE concentrations (1.0–2.0 mL·L^−1^) significantly enhanced plant growth, chlorophyll a, carotenoid levels, DPPH radical scavenging, and total flavonoid content relative to control. The 2.0 mL·L^−1^ treatment produced the highest total phenolic content (1.88-fold increase over the control) and was associated with elevated benzoic acid, rutin, quercetin, and kaempferol, along with reduced trans-cinnamic acid, indicating activation of the phenylpropanoid pathway. Moreover, all SE treatments significantly increased RA accumulation. These findings demonstrate that SE is an effective, sustainable biostimulant for Thai basil, with 2.0 mL·L^−1^ as the optimal concentration for maximizing growth and phytochemical production.

## 1. Introduction

Thai basil (*Ocimum basilicum* L.), a member of the Lamiaceae family, is a widely cultivated culinary and medicinal herb, especially popular in Southeast Asia [1,2]. Its distinctive aroma and flavor come from a rich composition of bioactive compounds, including phenolic acids, flavonoids, and essential oils such as linalool, methyl chavicol, and eugenol [3,4]. These compounds exhibit diverse biological activities, including antioxidant, antimicrobial, anti-inflammatory, and anticancer properties, making Thai basil valuable for the food, pharmaceutical, and cosmetic industries [5]. With the growing global demand for functional foods and natural health products, there is increasing interest in improving both the yield and phytochemical quality of basil [6,7]. However, the accumulation of these compounds is strongly affected by environmental factors and cultivation methods, highlighting the need for optimized production strategies to enhance its economic and nutritional value.

In recent years, plant factories with artificial lighting (PFALs) have become a cutting-edge approach for sustainable and high-quality herb production [8,9]. These systems create a fully controlled environment where key factors—such as temperature, humidity, light intensity, photoperiod, and nutrient supply—can be precisely regulated [9]. By eliminating external environmental fluctuations, PFALs enable consistent plant growth, improved uniformity, and year-round production of high-value crops [10]. To maximize the potential of PFALs, it is essential to complement environmental control with bio-based inputs that further stimulate plant performance.

Among such innovations, seaweed-based biostimulants have emerged as effective and eco-friendly solutions for improving plant growth, productivity, and stress resilience [11,12,13]. Derived primarily from brown algae such as *Ascophyllum nodosum* and *Sargassum* spp., these products contain diverse bioactive molecules—including phytohormones, polysaccharides, amino acids, and minerals—that regulate plant metabolism and enhance physiological efficiency [14]. Numerous studies have demonstrated that seaweed extracts (SE) can enhance photosynthetic activity, nutrient uptake, and root development while also stimulating the production of antioxidant enzymes and secondary metabolites [14,15,16]. Their application has shown positive effects across a wide range of crops and environments: enhanced yield and nutrient uptake in soybean under rainfed conditions [15], increased sucrose content and photosynthetic activity in sugarcane [17], and improved drought tolerance and flower development in *Hydrangea paniculata* [18]. In fruit crops such as peach and apricot, foliar applications of seaweed and yeast extracts have boosted fruit uniformity, polyphenol accumulation, and antioxidant capacity [16]. Similarly, leafy and microgreen vegetables like cabbage and arugula exhibited elevated phenolic and flavonoid contents after treatment with *Ascophyllum nodosum* extracts [19,20]. In various horticultural crops, foliar application of SE has led to measurable improvements in productivity and biochemical quality. For instance, treatments of 4.0–5.0 L ha^−1^ enhanced fruit uniformity and polyphenol accumulation in apricot [16], while similar formulations increased tomato yield by 4.6–6.9% through improved photosynthetic capacity [21]. In hot pepper, a 0.5% SE raised the yield to 4.23 kg m^−2^ and boosted total antioxidant activity by nearly 18% [22], and antioxidant values in tomato fruits rose by 38% (ABTS) and 11% (DPPH) after biostimulant treatment [23]. These findings underline the broad-spectrum potential of biostimulants to promote crop quality and sustainability in both open-field and controlled environments.

While seaweed-based biostimulants have been widely studied in field and greenhouse settings, their impact under fully controlled PFAL conditions—particularly for aromatic and medicinal plants such as Thai basil—remains limited. Therefore, this study aimed to evaluate how foliar application of seaweed-based biostimulants influences growth, antioxidant enzyme activities, and bioactive compound accumulation in Thai basil grown in a plant factory. The findings will provide valuable insights into sustainable, high-quality production systems for basil and other medicinal herbs.

## 2. Results

### 2.1. Growth Parameters

**Figure 1:**
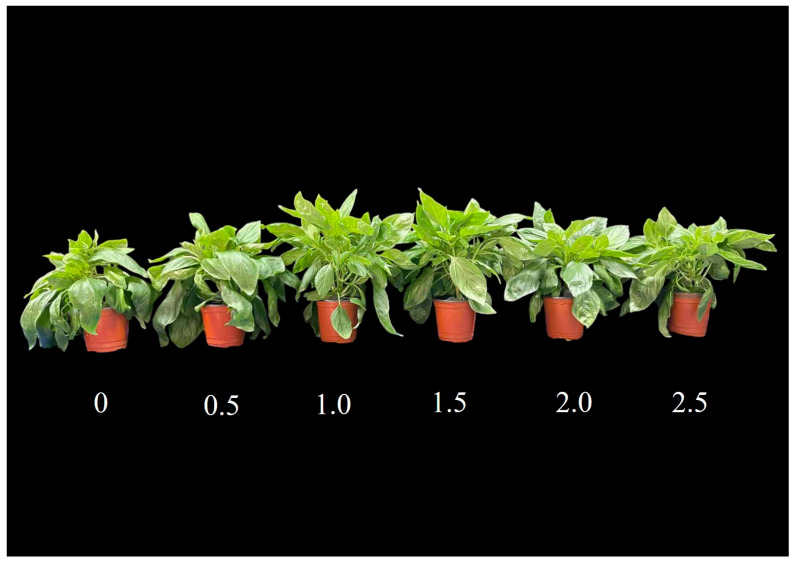
Growth performance of Thai basil plants under different seaweed extract treatments (0, 0.5, 1.0, 1.5, 2.0, and 2.5 mL·L^−1^) at four weeks after transplantation.

**Figure 2:**
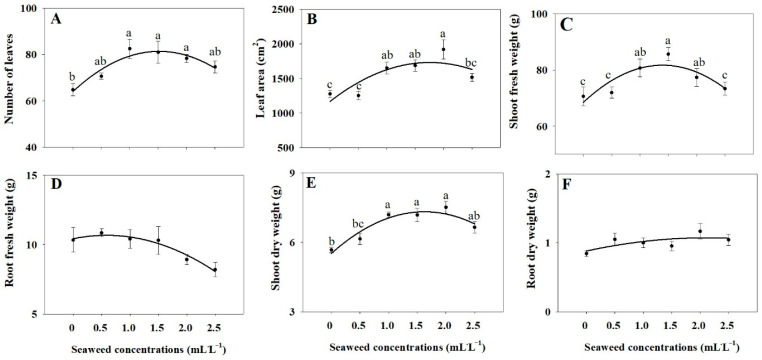
Number of leaves (**A**), leaf area (**B**), shoot fresh weight (**C**), root fresh weight (**D**), shoot dry weight (**E**), and root dry weight (**F**) of Thai basil under different seaweed extract treatments (0, 0.5, 1.0, 1.5, 2.0, and 2.5 mL·L^−1^). Values are means ± SE (n = 6). Different letters indicate statistically significant differences (*p* ≤ 0.05) based on ANOVA and Tukey’s post hoc test.

**Figure 3:**
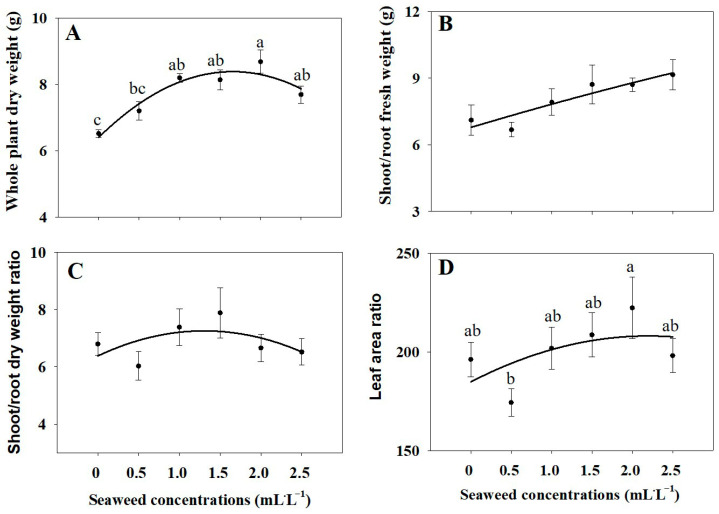
Whole plant dry weight (**A**), shoot/root fresh weight (**B**), shoot/root dry weight (**C**), and leaf area ratio (**D**) of Thai basil under different seaweed extract treatments (0, 0.5, 1.0, 1.5, 2.0, and 2.5 mL·L^−1^). Values are means ± SE (n = 6). Different letters indicate significant differences at *p* ≤ 0.05 based on ANOVA and Tukey’s test.

The results in Figure 1 and Figure 2 reveal that SE treatments of 1.0, 1.5, and 2.0 mL·L^−1^ significantly promoted leaf number, leaf area, and shoot biomass, both fresh and dry, compared with untreated controls (Figure 1 and Figure 2A–C,E). By contrast, there was no significant difference in root fresh and dry weights among control and SE treatments (Figure 2D,F). As shown in Figure 1 and Figure 3 the whole plant dry weight was significantly enhanced under the 1.0, 1.5, 2.0, 2.5 mL·L^−1^ SE treatments (Figure 3A) compared to the control, whereas the shoot-to-root fresh and dry weight ratios did not differ significantly among treatments, indicating balanced biomass allocation between shoots and roots (Figure 3B–D).

### 2.2. Chlorophyll, Carotenoids, and Total Flavonoid

**Figure 4:**
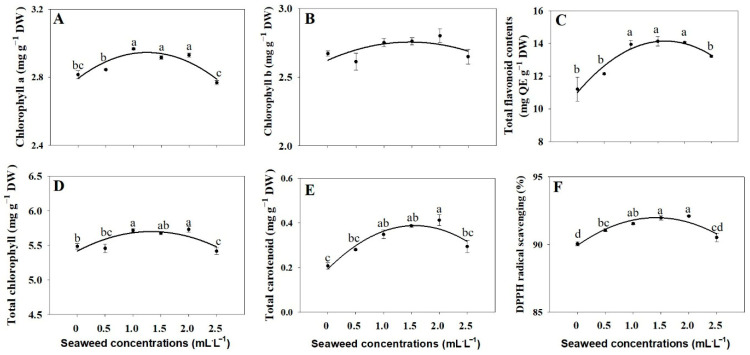
Chlorophyll a (**A**), chlorophyll b (**B**), total flavonoid contents (**C**), total chlorophyll (**D**), total carotenoid (**E**), and DPPH radical scavenging activity (**F**) of Thai basil under different seaweed extract treatments (0, 0.5, 1.0, 1.5, 2.0, and 2.5 mL·L^−1^). Values are means ± SE (n = 3). Different letters indicate significant differences at *p* ≤ 0.05.

Chlorophyll a (Chl a) levels were significantly higher in the 1.0, 1.5, and 2.0 mL·L^−1^ treatments compared with the untreated group (Figure 4A), while chlorophyll b (Chl b) remained unchanged (Figure 4B). The higher values for the total Chl were observed in the 1.0 and 2.0 mL·L^−1^ SE treatments group compared to the untreated group (Figure 4D). Significant increases in flavonoid content were observed in plants subjected to 1.0, 1.5, and 2.0 mL·L^−1^ SE treatments, compared to the untreated group (Figure 4C). The highest total carotenoid content was observed in the 2.0 mL·L^−1^ SE treatment, which was 1.98-fold higher than that of the control group (Figure 4E). A significant increase in DPPH radical scavenging was observed in the 0.5, 1.0, 1.5, and 2.0 mL·L^−1^ SE treatments (Figure 4F). Flavonoid levels and DPPH radical scavenging activity also increased significantly at moderate SE concentrations (1.0–2.0 mL·L^−1^), indicating enhanced antioxidant potential (Figure 4C,F).

### 2.3. Photosynthetic Characteristics

**Figure 5:**
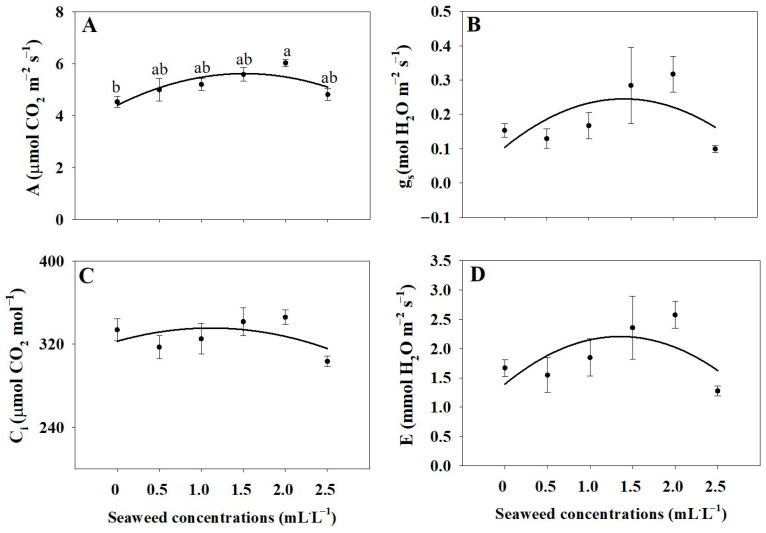
Net photosynthetic rate (A; **A**), stomatal conductance (gs; **B**), intercellular CO_2_ concentration (Ci; **C**), and transpiration rate (E; **D**) of Thai basil under different seaweed extract treatments (0, 0.5, 1.0, 1.5, 2.0, and 2.5 mL·L^−1^). Values are means ± SE (n = 3). Different letters indicate significant differences at *p* ≤ 0.05 as determined by one-way ANOVA followed by Tukey’s test.

The net photosynthetic rate was significantly higher under the 2.0 mL·L^−1^ treatment than in the control, indicating enhanced photosynthetic efficiency (Figure 5A). However, stomatal conductance, intercellular CO_2_ concentration, and transpiration rate were not significantly affected by SE treatments (Figure 5B–D), suggesting that improvements in photosynthesis were driven primarily by biochemical factors rather than changes in stomatal behavior.

### 2.4. Rosmarinic Acid and Total Phenolic Concentrations and Contents

The concentration of caffeic acid was slightly higher in the 2.5 mL·L^−1^ treatment compared to the control, though differences among treatments were not significant (Table 1). Benzoic acid content increased significantly in the 1.0, 1.5, and 2.0 mL·L^−1^ SE treatments relative to the control. Rutin concentrations were significantly higher in the 0.5, 1.0, 1.5, and 2.5 mL·L^−1^ SE treatments compared to the untreated group. In contrast, trans-cinnamic acid concentrations decreased significantly in all SE treatments compared to the untreated group. The highest quercetin concentration was recorded in the 1.5 mL·L^−1^ SE treatment, which was significantly greater than the control. Moreover, kaempferol levels increased significantly in the 1.5, 2.0, and 2.5 mL·L^−1^ SE treatments compared to the control (Table 1).

**Figure 6:**
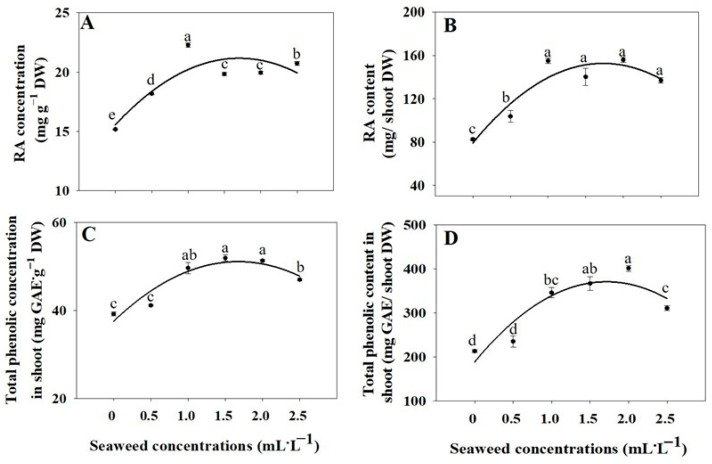
Rosmarinic acid (RA) concentration (**A**) and content (**B**), total phenolic concentration (**C**) and content (**D**) in basil shoot under different seaweed extract treatments (0, 0.5, 1.0, 1.5, 2.0, and 2.5 mL·L^−1^). Values are means ± SE (n = 3). Different letters indicate significant differences at *p* ≤ 0.05 using ANOVA followed by Tukey’s test.

Significant increases in RA concentrations and contents were observed in plants subjected to all SE treatments, compared to the control (Figure 6A,B). Exposure to seaweed extract for 1.0, 1.5, 2.0, and 2.5 mL·L^−1^ resulted in a significant elevation of total phenolic concentrations and contents in shoot (Figure 6C,D). The higher levels of RA content were observed with the 1.0, 1.5, 2.0, and 2.5 mL·L^−1^ SE treatments compared to the untreated group. The highest levels of total phenolic content were observed with the 2 mL·L^−1^ SE treatment, showing increases of 1.88-fold, respectively, compared to the control.

## 3. Discussion

### 3.1. Plant Growth Parameters

Seaweed extract (SE), especially at 1.0–2.0 mL·L^−1^, significantly boosted basil growth by increasing leaf number, leaf area, and shoot biomass, likely due to natural hormones like auxins and cytokinins in the SE [14]. These compounds stimulate cell division and elongation, enhance bud initiation, and improve chloroplast development, which collectively boost photosynthetic efficiency and shoot biomass accumulation [24,25]. Additionally, the extract enhances nutrient uptake and activates antioxidant defense systems, allowing plants to better tolerate stress and allocate more resources to shoot growth [13,26]. Consistent with previous studies on other crops, the SE markedly promoted tomato plant growth by increasing flower and fruit numbers, enhancing flower cluster development, extending root length, boosting root and shoot dry weight, and raising SPAD values, while simultaneously improving fruit quality and overall yield [27]. SE application increased tomato yield by 4.6–6.9%, with the 60 kg hm^−2^ (=60 kg per hectare) treatment achieving the highest yield through enhanced photosynthesis [21]. Similar growth-promoting effects were observed in peppermint and purple basil, where SE enhanced leaf biomass and overall plant vigor [28]. In crops such as soybean and spinach, SE treatment improved vegetative growth, leaf chemical composition, and antioxidant activity, suggesting that the stimulatory effect of SE may be generalizable across multiple plant species [15,29]. The foliar application of 7.5 mL·L^−1^ SE resulted in optimal biomass yield, growth, and essential oil content and composition in holy basil [30]. Seaweed application significantly enhanced all measured traits, except for flower length and stem diameter [31]. Biostimulant treatments increased plant fresh weight, leaf number, and plant height in basil compared to the control [32]. In our study, although the whole-plant dry weight significantly increased at concentrations of 1.0 to 2.5 mL·L^−1^, the shoot-to-root fresh and dry weight ratios did not change significantly among treatments. This indicates that both shoot and root biomasses increased proportionally, reflecting balanced whole-plant growth rather than preferential biomass allocation to a specific organ. The foliar application of seaweed extract likely exerted its main effects on above-ground tissues, while root growth was already optimized under hydroponic conditions. Similar observations were reported by Sleight et al. (2023) [33], who found that although the distribution of below-ground biomass varied among willow cultivars and sites, the root-to-shoot ratio did not differ significantly across sites (*p* = 0.8970), cultivars (*p* = 0.2834), or their interaction (*p* = 0.8481). At the highest concentration (2.5 mL·L^−1^), growth did not increase further, possibly due to hormonal imbalance or mild phytotoxic effects. Vargas-Hernández et al. (2017) reported that excessive biostimulant concentrations can cause hormonal or metabolic disturbances that suppress plant growth [34]. Similarly, Arun et al. (2014) observed that increasing seaweed extract concentrations beyond optimal levels inhibited seed germination and growth in *Solanum lycopersicum* due to toxicity effects [35].

Overall, these findings demonstrate that moderate SE concentrations, especially 1.5–2.0 mL·L^−1^, effectively enhance basil growth by promoting shoot development and overall plant vigor without negatively impacting root growth. Thus, moderate foliar application of SE can maximize shoot development without compromising root growth in hydroponic systems.

### 3.2. Chlorophyll and Total Carotenoid Levels

The results showed that seaweed extract had a strong positive effect on basil growth and physiology at moderate concentrations. Treatments with 1.0, 1.5, and 2.0 mL·L^−1^ SE significantly increased chlorophyll a (Chl a) content compared to the control, likely due to the presence of natural plant hormones such as cytokinins and auxins reported by the manufacturer to be contained in the commercial SE product, which stimulate chloroplast development and chlorophyll biosynthesis, thereby enhancing photosynthetic efficiency [13,36]. In contrast, chlorophyll b (Chl b) levels did not change significantly, as this pigment mainly functions in light harvesting and remains relatively stable under optimal growing conditions. Total chlorophyll was highest in the 1.0 and 2.0 mL·L^−1^ treatments, primarily driven by the increase in Chl a. The total carotenoid content reached its peak at 2.0 mL·L^−1^, nearly doubled relative to untreated plants, indicating improved photoprotection and antioxidant defense. Similarly, SE application increased chlorophyll and carotenoid levels in peppermint, arugula, and spinach, suggesting that the enhancement of photosynthetic pigments by algae-based biostimulants is a broadly conserved response [20,28,29]. Similarly, flavonoid content was significantly elevated at 1.0, 1.5, and 2.0 mL·L^−1^, which may be attributed to the elicitor-like activity of seaweed extracts that can enhance the biosynthesis of antioxidant compounds such as flavonoids [13,37]. In agreement with previous studies, flavonoid content in cabbage increased under SE treatment compared to the control [19]. This was supported by the marked increase in DPPH radical scavenging activity observed in the 0.5–2.0 mL·L^−1^ treatments, reflecting higher antioxidant capacity in the treated plants. Similar increases in flavonoid accumulation and antioxidant activity have been reported in sweet basil, mint, and spinach following SE application, suggesting that SE consistently enhances secondary metabolite production across plant species [29,38]. Consistent with earlier reports, the highest rise in flavonoid content (89%) and DPPH antioxidant activity (82%) in arugula occurred at a 2.5% biostimulant concentration compared to the control [20]. Overall, these findings suggest that SE acts as an effective biostimulant, enhancing photosynthesis, secondary metabolite production, and antioxidant defense in basil, with 1.0–2.0 mL·L^−1^ being the optimal range for maximum benefits, while higher concentrations may not provide additional improvements due to nutrient saturation or slight phytotoxic effects.

### 3.3. Photosynthetic Parameters

The application of SE at 2.0 mL·L^−1^ significantly enhanced the net photosynthetic rate (A) in basil compared to the control, likely due to the presence of natural plant growth regulators such as cytokinins and auxins, as well as essential nutrients found in SE. These compounds promote chloroplast development, stimulate chlorophyll biosynthesis, and improve the efficiency of the photosynthetic machinery, leading to greater CO_2_ fixation and higher photosynthetic capacity [28,39,40]. Studies in sugarcane and tomato also reported increased photosynthetic rates following SE application, confirming that the biochemical stimulation of photosynthesis by algae-derived biostimulants is a common mechanism across different plant species [17,21]. In contrast, stomatal conductance (gs), intercellular CO_2_ concentration (Ci), and transpiration rate (E) did not show significant differences among treatments. This suggests that the improvement in photosynthesis was primarily due to biochemical and physiological enhancements within the leaf tissue, rather than changes in stomatal behavior or water regulation. Since SE was applied foliarly and the plants were grown under controlled hydroponic conditions with optimal water availability, stomatal activity remained stable, while internal biochemical processes drove the observed increase in photosynthetic performance [18]. In controlled hydroponic systems, stable stomatal behavior helps maintain efficient photosynthesis and water use, as plants are continuously supplied with adequate water and nutrients.

### 3.4. Rosmarinic Acid and Total Phenolic Concentrations and Contents

The increase in rosmarinic acid (RA) and total phenolic content in basil under seaweed extract (SE) treatments can be attributed to the bioactive compounds present in SE, such as natural plant growth regulators (auxins, cytokinins, and gibberellins), amino acids, trace minerals, and vitamins [30,38]. These compounds stimulate the phenylpropanoid pathway by enhancing the activity of key enzymes like phenylalanine ammonia-lyase, which promotes the biosynthesis of RA and other phenolic compounds [13,41]. Similar effects have been observed in arugula, spinach, and yarrow, where SE application enhanced phenolic accumulation, flavonoid content, and antioxidant activity, indicating a conserved mechanism for the stimulation of secondary metabolism by SE across different plant species [20,29,42]. As a result, plants treated with higher SE concentrations, particularly 2.0 mL·L^−1^, showed a substantial increase total phenolics, reaching 1.88-fold higher levels than the control. The increase in benzoic acid and rutin suggests an upregulation of early and intermediate steps of the phenylpropanoid pathway, while the decrease in trans-cinnamic acid indicates its rapid conversion into downstream metabolites [43]. Additionally, the significant rise in quercetin and kaempferol concentrations reflects an enhanced flavonoid biosynthesis and antioxidant defense. Lower concentrations of SE (0.5 and 1.0 mL·L^−1^) provided insufficient bioactive compounds to fully activate these metabolic pathways, whereas very high concentrations (2.5 mL·L^−1^) may have caused feedback regulation or metabolic diversion. Overall, the results demonstrate that foliar application of SE stimulates secondary metabolism and phenolic accumulation in basil, with 2.0 mL·L^−1^ being the optimal concentration for maximizing total phenolic production. These findings align with previous studies showed that foliar application of seaweed extract significantly enhanced vegetative growth, seed yield components, and leaf chemical composition (including sugars, proteins, amino acids, antioxidant activity, and volatile oil), as well as leaf and stem structure in basil. The most pronounced effects were observed at 1.5 mL·L^−1^ [44]. Applying 9 mL·L^−1^ seaweed extract boosted leaf phenolics and antioxidant activity by up to 30.44% over the control, improving yarrow’s medicinal quality [42]. SE treatments enhanced total phenols and ascorbic acid, thereby improving the functional quality of spinach [29]. Overall, these findings support a general mechanism in which SE foliar application stimulates phenylpropanoid metabolism, flavonoid biosynthesis, and antioxidant defense in diverse plant species.

## 4. Materials and Methods

### 4.1. Plant Material and Seedling Conditions

Thai basil (*Ocimum basilicum* Linn.) seeds sourced from Namheung Seed Co., Ltd. (Seoul, Republic of Korea) were sown in rockwool cubes and cultivated in a controlled environment chamber maintained at 21.2 ± 2 °C with 71 ± 10% relative humidity. The plants were exposed to LED lighting (TL5 14W/865, Philips, Amsterdam, The Netherlands) delivering a photosynthetic photon flux density (PPFD) of 200 ± 10 µmol·m^−2^·s^−1^ on a 16 h light/8 h dark cycle. Fourteen days after sowing, Thai basil seedlings were soaked in Hoagland solution with a pH of 6.6 and an EC of 1.04 dS·m^−1^.

### 4.2. Treatments and Experimental Design

At 28 days after sowing, basil seedlings were transferred to a plant factory and grown using a deep flow technique hydroponic system, with environmental conditions maintained as during the seedling stage. Each treatment consisted of 10 plants per replicate. The plants were cultivated for 28 days using Hoagland solution maintained at a pH of approximately 6.7 and an EC of 2.0 dS·m^−1^. To evaluate the effects of seaweed extract (SE), five concentrations (0.5, 1.0, 1.5, 2.0, and 2.5 mL·L^−1^) were applied twice per week via foliar spraying, while distilled water was used as the control. Each spraying was performed until all leaf surfaces were thoroughly wetted. The SE used in this study was a commercial product (Living Farm, K-INGS Co., Ltd., Busan, Republic of Korea) and was not prepared by the authors. According to the manufacturer’s label, this product is a liquid compound fertilizer containing SE derived from *Ascophyllum nodosum*, with 5% nitrogen (N), 3% available phosphoric acid (P_2_O_5_), 2% soluble potassium (K_2_O), 0.05% boron (B), and 0.0005% molybdenum (Mo). The SE also contains natural plant hormones such as auxins and cytokinins, which promote root growth, nutrient uptake, photosynthesis, and stress tolerance, while activating antioxidant enzymes. The experiment was arranged using a completely randomized design with two replicates.

### 4.3. Growth Parameter Measurements

Twenty-eight days after transplanting, leaf area, leaf number, and the fresh weights of shoots and roots were recorded with an electronic scale (EW220-3NM, Kern & Sohn GmbH., Balingen, Germany). Afterward, the samples were oven-dried at 70 °C for seven days to determine the dry weights (DW). Leaf area was measured with a leaf area meter (LI-3000A, Li-Cor, Lincoln, NE, USA). The leaf area ratio, shoot-to-root DW ratio, and shoot-to-root FW ratio were then calculated, with six plants used per replicate (n = 6).

### 4.4. Photosynthetic Characteristics

Photosynthetic parameters were measured on three plants (n = 3) per replicate using a LI-COR 6400 system (LI-COR Inc., Lincoln, NE, USA) under fixed chamber settings (400 μmol mol^−1^ CO_2_, 300 μmol m^−2^ s^−1^ PPFD, 25 °C, 500 cm^3^ s^−1^ airflow). Recorded variables included photosynthetic rate, stomatal conductance, CO_2_ concentration, and transpiration rate.

### 4.5. Photosynthetic Pigment Analysis

After harvest, basil shoots were rapidly frozen in liquid nitrogen and transferred within four days to a –50 °C dry freezer (TFD5503, IL Shinbiobase Co., Ltd., Dongducheon, Republic of Korea). Samples were ground with a porcelain mortar and pestle, sieved, and analyzed for chlorophyll (Chl) a, Chl b, and total cartotenoid, using an Epoch microplate spectrophotometer (EMS) (EPOCH-SN; Agilent Technologies, Inc., Santa Clara, CA 95051, USA). Powdered samples (20 mg DW) were extracted with 2 mL of 90% MeOH, centrifuged at 1308× *g* for 20 min, and absorbance was measured at 665.2, 652.4, and 470 nm for Chl a, Chl b, and Car, respectively, following Lichtenthaler, 1987 and Lam et al., 2023a [45,46]. Pigment concentrations were calculated as follows:

Chl a (mg·g^−1^) = (16.82 × A_665.2_ − 9.28 × A_652.4_)/10

Chl b (mg·g^−1^) = (36.92 × A_652.4_ − 16.54 × A_665.2_)/10

Total carotenoid (mg·g^−1^) = ([1000 × A_470_ − 1.91 × Chl a − 95.15 Chl b]/225)/10

Total Chl a + b (mg·g^−1^) = Chl a + Chl b

where A is the absorbance at wavelength.

### 4.6. DPPH Radical Scavenging Activity Analysis

DPPH radical scavenging activity was measured at 517 nm using an EMS, following Rahman Et Al., 2015 and Lam Et Al., 2023 [46,47]. DPPH activitiy was calculated as:

DPPH (%) = (A_blank_ − A_sample_)/A_blank_ × 100

where A is the absorbance at 517 nm.

### 4.7. Total Flavonoid Content

Total flavonoid content in basil plants was determined using the aluminum chloride (AlCl_3_) colorimetric method of Lin and Tang (2007) [48] method, with 20 mg of dry sample extracted in 2 mL of 90% methanol and sonicated at 20 °C for 20 min. The mixture was then centrifuged at 1308× *g* for 20 min at 4 °C. A 100 μL aliquot of the sample was mixed with 300 μL of 95% ethanol, 20 μL of 10% AlCl_3_, 20 μL of 10% potassium acetate, and 600 μL of triple-distilled water. The absorbance was measured at 415 nm after 40 min of incubation at room temperature at 22 °C using an EMS. Total flavonoid content was determined in triplicate (n = 3) using quercetin (QE) as the standard, with a calibration curve prepared from quercetin solutions of 0, 50, 75, 125, 250, and 500 μg/mL in methanol, and the results expressed as mg QE/g DW.

### 4.8. Phenolic Compound Analysis by HPLC

Phenolic constituents in basil were quantified using a 1260 Infinity Series High-performance liquid chromatography (HPLC) system (1260 Infinity; Agilent Technologies Inc., Santa Clara, CA, USA) [49,50]. Dry powder (100 mg) was extracted with 2 mL of 80% methanol, sonicated for 1 h with intermittent vortexing, centrifuged (12,000× *g*, 10 min), and filtered (0.45 µm). Analysis was performed by HPLC on a Symmetry C18 column (30 °C) using 0.15% acetic acid in water (solvent A) and methanol (solvent B) at 1.0 mL·min^−1^, with UV detection at 280 nm. The 98 min gradient of solvent B was: 5% (0–1 min), 15% (1–9 min), 20% (9–24 min), 30% (24–54 min), 45% (54–66 min), 56% (66–76 min), 60% (76–80 min), 80% (80–91 min), and 5% (91–98 min). External standards (100–500 µg·mL^−1^; Sigma-Aldrich, Co., Ltd., Seoul, Republic of Korea) of caffeic acid, benzoic acid, rutin, trans-cinnamic acid, quercetin, and kaempferol were used to construct calibration curves (R^2^ ≥ 0.9999). Retention times were 27.59, 56.81, 61.75, 72.34, 75.13, and 82.08 min, respectively. Each sample was analyzed in triplicate with an injection volume of 20 µL.

### 4.9. Determination of Rosmarinic Acid

Rosmarinic acid (RA) content was determined following Lam Et Al. (2024) [51]. Samples were frozen in liquid nitrogen, freeze-dried at −50 °C for four days, ground, and sieved. Dry powder (200 mg) was extracted with 10 mL methanol, sonicated for 30 min, centrifuged (1358× *g*, 20 min), and filtered (0.45 µm). HPLC was performed using 0.1% formic acid in water (solvent A) and acetonitrile (solvent B) under the following gradient: 20% B (0–5 min), 50% B (5–20 min), and 100% B (20–22 min). The flow rate was 0.8 mL·min^−1^ with 10 µL injection and detection at 330 nm. Standard RA (Sigma-Aldrich, Co., Ltd., Seoul, Republic of Korea) showed a retention time of 11.655 min, with calibration linearity (R^2^ = 0.9999) across 0.1–100 µg·mL^−1^ (LOD = 0.06 µg·mL^−1^; LOQ = 0.20 µg·mL^−1^). The RA content of shoots was determined by multiplying RA concentration (mg g^−1^ DW) by shoot DW (g).

### 4.10. Statistical Analysis

Growth parameters were measured in six plants (n = 6), and three plants (n = 3) per replicate were used for physiological and biochemical analyses, plants were randomly selected from each replicate. The experiment used a completely randomized design with two replicates. Data were analyzed by one-way ANOVA and Tukey’s test (*p* < 0.05) using SPSS version 20.0 (IBM Corp., Armonk, NY, USA).

## 5. Conclusions

Foliar application of Ascophyllum nodosum-derived SE significantly improved the growth, photosynthetic performance, and phytochemical quality of Thai basil cultivated in a fully controlled plant factory. Moderate concentrations (1.0–2.0 mL·L^−1^) consistently enhanced leaf area, shoot biomass, chlorophyll a, carotenoids, flavonoids, and antioxidant capacity. The 2.0 mL·L^−1^ treatment yielded the greatest increases in total phenolics and rosmarinic acid, approximately 1.9-fold higher than in untreated plants. The concurrent rise in key phenolics (benzoic acid, rutin, quercetin, kaempferol) and the reduction in trans-cinnamic acid indicate activation of the phenylpropanoid pathway. Higher concentrations provided no additional benefit, establishing 2.0 mL·L^−1^ as the practical optimum for maximizing both biomass and phytochemical quality without affecting root allocation. These findings demonstrate that A. nodosum-based SE is a sustainable and effective biostimulant for premium basil production in plant factory systems. Future research should refine application strategies across cultivars and growth stages and employ metabolomic and transcriptomic approaches to better link biochemical improvements with sensory attributes and postharvest quality.

## Figures and Tables

**Table 1:** Phenolic compounds in basil under different seaweed treatments (0-, 0.5-, 1.0-, 1.5-, 2.0-, and 2.5- mL·L^−1^. Values represent means (n = 3).

^w^ SE Concentration Treatments (mL·L^−1^)	Phenolic Compounds in Basil (mg·g^−1^ DW)					
	Caffeic Acid (mg·g^−1^ DW)	Benzoic Acid (mg·g^−1^ DW)	Rutin (mg·g^−1^ DW)	Trans-Cinnamic Acid (mg·g^−1^ DW)	Quercetin (mg·g^−1^ DW)	Kaempferol (mg·g^−1^ DW)
0	0.42 ± 0.002 ab	6.04 ± 0.475 d	32.17 ± 0.266 d	0.0159 ± 0.00019 a	0.159 ± 0.0004 b	0.395 ± 0.0040 c
0.5	0.35 ± 0.005 c	5.75 ± 0.208 d	34.55 ± 0.020 c	0.0067 ± 0.00011 b	0.152 ± 0.00005 c	0.335 ± 0.0066 d
1.0	0.34 ± 0.008 c	9.45 ± 1.228 c	39.35 ± 0.531 a	0.0039 ± 0.00026 b	0.146 ± 0.0005 d	0.383 ± 0.0044 c
1.5	0.40 ± 0.496 b	13.78 ± 0.516 b	37.14 ± 0.355 b	0.0095 ± 0.00293 b	0.174 ± 0.0004 a	0.435 ± 0.0042 b
2.0	0.42 ± 0.005 ab	16.99 ± 0.100 a	33.30 ± 0.147 cd	0.0086 ± 0.00038 b	0.147 ± 0.0016 d	0.461 ± 0.0022 a
2.5	0.43 ± 0.002 a	6.09 ± 0.561 d	39.88 ± 0.401 a	0.0077 ± 0.00001 b	0.144 ± 0.0004 d	0.455 ± 0.0048 ab
Significance ^z^	***	***	***	***	***	***

^w^ SE concentration; ^z^ significant at *** *p* ≤ 0.001. Values are means ± SE (n = 3). Different letters in a column indicate significant differences among treatments at *p* < 0.05 (Tukey’s test), DW = dry weight.

## Data Availability

The original contributions are included in the article. Further queries can be directed to the corresponding author.
